# Characterization of symptoms and determinants of disease burden in dementia with Lewy bodies: DEvELOP design and baseline results

**DOI:** 10.1186/s13195-021-00792-w

**Published:** 2021-02-26

**Authors:** M. van de Beek, I. van Steenoven, J. J. van der Zande, I. Porcelijn, F. Barkhof, C. J. Stam, P. G. H. M. Raijmakers, P. Scheltens, C. E. Teunissen, W. M. van der Flier, A. W. Lemstra

**Affiliations:** 1 Alzheimer Center Amsterdam, Department of Neurology, Amsterdam Neuroscience, Vrije Universiteit Amsterdam, Amsterdam UMC, Amsterdam, The Netherlands; 2 Department of Radiology and Nuclear Medicine, Amsterdam Neuroscience, Amsterdam UMC, Amsterdam, The Netherlands; 3 Institutes of Neurology and Healthcare Engineering, UCL, London, England, UK; 4 Department of Clinical Neurophysiology and MEG center, Vrije Universiteit Amsterdam, Amsterdam UMC, Amsterdam, The Netherlands; 5 Neurochemistry, Department of Clinical Chemistry, Amsterdam Neuroscience, Vrije Universiteit Amsterdam, Amsterdam UMC, Amsterdam, The Netherlands; 6 Department of Epidemiology and Data Sciences, Vrije Universiteit Amsterdam, Amsterdam UMC, Amsterdam, The Netherlands

**Keywords:** Dementia with Lewy bodies, Clinical manifestation, Disease burden, Dementia, Quality of life, Caregiver burden, IADL

## Abstract

**Background:**

The DEmEntia with LEwy bOdies Project (DEvELOP) aims to phenotype patients with dementia with Lewy bodies (DLB) and study the symptoms and biomarkers over time. Here, we describe the design and baseline results of DEvELOP. We investigated the associations between core and suggestive DLB symptoms and different aspects of disease burden, i.e., instrumental activities of daily living (IADL) functioning, quality of life (QoL), and caregiver burden.

**Methods:**

We included 100 DLB patients (69 ± 6 years, 10%F, MMSE 25 ± 3) in the prospective DEvELOP cohort. Patients underwent extensive assessment including MRI, EEG/MEG, ^123^FP-CIT SPECT, and CSF and blood collection, with annual follow-up. Core (hallucinations, parkinsonism, fluctuations, RBD) and suggestive (autonomous dysfunction, neuropsychiatric symptoms) symptoms were assessed using standardized questionnaires. We used multivariate regression analyses, adjusted for age, sex, and MMSE, to evaluate how symptoms related to the Functional Activities Questionnaire, QoL-AD questionnaire, and Zarit Caregiver Burden Interview.

**Results:**

In our cohort, RBD was the most frequently reported core feature (75%), while visual hallucinations were least frequently reported (39%) and caused minimal distress. Suggestive clinical features were commonly present, of which orthostatic hypotension was most frequently reported (64%). Ninety-five percent of patients showed EEG/MEG abnormalities, 88% of ^123^FP-CIT SPECT scans were abnormal, and 53% had a CSF Alzheimer’s disease profile. Presence of fluctuations, lower MMSE, parkinsonism, and apathy were associated with higher IADL dependency. Depression, constipation, and lower IADL were associated with lower QoL-AD. Apathy and higher IADL dependency predisposed for higher caregiver burden.

**Conclusion:**

Baseline data of our prospective DLB cohort show clinically relevant associations between symptomatology and disease burden. Cognitive and motor symptoms are related to IADL functioning, while negative neuropsychiatric symptoms and functional dependency are important determinants of QoL and caregiver burden. Follow-up is currently ongoing to address specific gaps in DLB research.

**Supplementary Information:**

The online version contains supplementary material available at 10.1186/s13195-021-00792-w.

## Introduction

Dementia with Lewy bodies (DLB) is the second most common form of neurodegenerative dementia in the elderly after Alzheimer’s disease (AD), affecting 4–10% of all dementia cases [1, 2]. Despite the high prevalence of DLB, research lags behind when compared to AD, and there are unmet needs in understanding disease manifestation and progression. DLB is defined by cognitive decline accompanied by visual hallucinations, parkinsonism, cognitive fluctuations, and/or REM sleep behavior disorder (RBD). Suggestive features include autonomic symptoms, such as orthostatic hypotension and constipation, and neuropsychiatric symptoms, such as depression, apathy, anxiety, and delusions [3]. Not all patients develop all symptoms along the course of the disease, and there is clinical and pathological overlap with both AD and Parkinson’s disease, making DLB a complex disease, both in terms of pathology, diagnosis, and disease management.

Disease burden in DLB is high, with lower quality of life (QoL) [4, 5], higher instrumental activities of daily living (IADL) dependency [6], and higher caregiver burden [7, 8] compared to AD. Which DLB characteristics and symptoms contribute to these measures of disease burden has not yet been fully elucidated. Understanding these contributions is relevant for the development of outcome measures for trials and treatment. Although no curative therapy exists, symptomatic treatment for the various symptoms of DLB is available [9]. Treating DLB patients, however, is challenging due to the considerable side effects of medication. There is a paucity of evidence from clinical trials specific to DLB. In view of a personalized approach, it is necessary to understand how symptoms affect disease burden.

To contribute to the specific gaps in the knowledge of clinical presentation, biological mechanisms, biomarker development, progression, and predictive factors, we initiated the DEmentia with LEwy BOdies Project (DEvELOP) cohort. With this observational cohort, we aimed to characterize DLB patients and identify biological and clinical prognostic factors for the decline. In the current paper, we present the study design and baseline results of DEvELOP. We provide an in-depth characterization of DLB symptoms and cross-sectionally evaluate which clinical symptoms contribute to different aspects of disease burden.

## Methods

### Study design

DEvELOP is a longitudinal prospective cohort study embedded in the Amsterdam Dementia Cohort (ADC). All patients in the ADC were referred to our memory clinic for analysis of cognitive complaints by their local specialist or general practitioner. Patients underwent a 1-day standardized screening, which included medical history; physical, neurological, and neuropsychological examinations; magnetic resonance imaging (MRI); electroencephalography (EEG) or magnetoencephalography (MEG); and blood and CSF collection [10]. Diagnoses were made in a multidisciplinary meeting.

The inclusion criteria for DEvELOP are a diagnosis of possible or probable DLB [3, 11] or a diagnosis of mild cognitive impairment with at least two DLB core features or one feature with an abnormal ^123^FP-CIT-SPECT (MCI-LB) [12, 13]. All MCI-LB patients fulfilled the recently published research criteria for MCI-LB [14]. For inclusion, patients had to have a Clinical Dementia Rating (CDR) of 0.5 or 1 and MMSE ≥18. The exclusion criteria were severe physical or life-threatening conditions and nursing home admittance. ^123^FP-CIT-SPECT scans were made at the discretion of the treating physician, to assure diagnostic accuracy. Other indicative biomarkers for DLB (polysomnography and ^123^iodine-MIBG myocardial scintigraphy) were not collected. The DEvELOP protocol follows the JPND guidelines for prospective cohort studies in DLB, in order to facilitate data sharing [15].

Eligible patients were informed about the study and invited to participate. Additional baseline measures were assessed during a baseline visit. All patients are invited for annual follow-up, which consists of clinical examination, physical examination, extensive neuropsychological assessment, and questionnaires. At 2 years of follow-up, patients are invited for repeated MRI and lumbar puncture. Inclusion of patients started in March 2016, and we included a total of 100 patients. The local medical ethics committee approved the study, and patients and caregivers gave their written informed consent. The research is performed according to the principles of the Declaration of Helsinki.

### Clinical measurements

#### Cognitive symptoms and neuropsychological assessment

Global cognition was estimated with the Clinical Dementia Rating (CDR), MMSE, and MoCa. Duration of complaints was systematically assessed during the patient history interview and was defined as the years of complaints before the baseline visit. All patients received standardized neuropsychological assessment as part of the routine memory clinic evaluation with additional tests as part of the DEvELOP baseline visit (Supplementary Table 1). All tests were performed at baseline and are repeated at follow-up. For this paper, we created cognitive domain scores from individual neuropsychological tests. Memory was assessed with the visual association test (VAT) and the immediate recall and delayed recall of the Dutch version of the verbal learning test [16, 17]. For attention, we used Trail Making Test part A (TMT-A), Stroop test parts 1 and 2, and forward condition of the digit span (extended version) [18–20]. Executive functions were measured with the score of Trail Making Test part B controlled for the score on part A (TMT-B/A), the score of Stroop part 3 controlled for the score on part 2 (Stroop 3/2), digit span backwards (extended version), letter fluency, and the Frontal Assessment Battery (FAB) [19–22]. Three subtests of the VOSP battery for visual-spatial functioning (number-location test, dot counting, and fragmented letters) were used to assess visual-spatial functioning [23]. Language was assessed with the Visual Association Naming Task (VAT-naming) and category fluency [16, 21]. We calculated the inverse scores for time-dependent tests, so that higher scores equaled better performance. Neuropsychological data were converted to *z*-scores, using baseline data of an independent group of cognitively healthy subjects (*n* = 533, 60 ± 10 years, 54%F, MMSE = 29 ± 1).

#### Core and suggestive clinical features

Parkinsonism was assessed using the Unified Parkinson’s Disease Rating Scale (UPDRS) motor scale (range 0–108) [24]. The Hoehn and Yahr Scale (H&Y) was used to stage the level of parkinsonism [25]. RBD was assessed with the Mayo Sleep Questionnaire (MSQ) [26]. Fluctuating cognition was assessed with the Clinical Assessment of Fluctuations (CAF, range 0–16) [27] and the Mayo Fluctuations Questionnaire (MFQ) [28]. Hallucinations were assessed with the Questionnaire on Psychotic Experiences (QPE), which is an extensive clinical questionnaire that assesses the severity, frequency, and content of hallucinations in all modalities and delusions [29]. The QPE was assessed during an interview with the patient and their caregiver. Hallucinations (visual, auditory, other modalities) were rated on frequency, duration, distress, and impact on a 6-item scale, total score per modality ranging from 0 to 24. Furthermore, qualitative questions assessed phenomenological characteristics of the hallucinations, such as the nature and content of hallucinations.

Other neuropsychiatric symptoms, referred to as supportive symptoms in the 2017 DLB-criteria, included depression, delusions, apathy, and anxiety. For depressive symptoms, we used the total score of the Geriatric Depression Scale (GDS, range 0–15), with higher scores indicating more depressive symptoms [30]. The QPE delusions assessed the presence of nine common types of delusions: paranoid, reference, guilt, control, religious, grandeur, somatic, Cotard’s syndrome (the belief that the person has died), and Capgras syndrome (the belief that a known person has been replaced by an impostor). Apathy and anxiety were assessed with the apathy and anxiety subscores of the 12-item Neuropsychiatric Inventory (NPI) [31].

Supportive autonomic dysfunctions include orthostatic hypotension, constipation, and micturition problems. For orthostatic hypotension (OH), blood pressure (BP) was measured three times, once lying down, followed by a BP measurement at 1 and 3 min after standing. OH was defined as having a ≥ 20 mmHg drop in systolic BP (SBP) or a ≥ 10 mmHg drop in diastolic BP (DBP) at 1 and/or 3 min after standing [32]. Constipation and urinary problems were assessed using the Non-Motor Symptoms Scale (NMSS) [33].

#### Cutoffs for symptom presence

For analyses, the presence of core and suggestive symptoms was dichotomized into being present or not. Operationalization and cutoff values for the determinants and outcomes in analyses are displayed in Table 1.

**Table 1:** Operationalization of core and suggestive symptoms and measures of disease burden

Symptom domain	Symptom	Operationalization and cutoff
Core clinical features	Visual hallucinations	QPE visual hallucinations ≥1
	Parkinsonism	UPDRS: ≥1 on bradykinesia, with ≥1 on rigidity and/or ≥1 on resting tremor subscores
	RBD	Mayo sleep questionnaire ≥1
	Cognitive fluctuations	Clinical assessment of fluctuations ≥5
Suggestive neuropsychiatric symptoms	Depressive symptoms	Geriatric Depression Scale ≥6
	Apathy	NPI apathy subscale ≥1
	Anxiety	NPI anxiety subscale ≥1
	Delusions	QPE delusions ≥1
Suggestive autonomic symptoms	Orthostatic hypotension	≥20 mmHG drop in SBP or ≥10 mmHG drop in DBP
	Constipation	NMSS constipation question (Q21) ≥1
	Micturinal problems	NMSS micturinal question (Q22) ≥1
Assessment of disease burden	IADL	Functional Activities Questionnaire (0–30)
	Quality of life	Quality of Life-AD [13–52]
	Caregiver burden	Zarit Caregiver Burden Interview (0–88)

*Abbreviations*: *QPE* questionnaire on psychotic experiences, *UPDRS* Unified Parkinson’s Disease Rating Scale, *RBD* rapid eye movement behavior disorder, *NPI* Neuropsychiatric Inventory, *SBP* systolic blood pressure, *DBP* diastolic blood pressure, *NMSS* Non-Motor Symptoms Scale, *IADL* instrumental activities of daily living

#### Assessment of disease burden

We assessed disease burden on three outcomes: IADL performance, QoL, and caregiver burden. The Functional Activities Questionnaire (FAQ) was used to assess IADL performance (range 0–30) [34]. Caregivers rated 10 categories of IADL, such as keeping financial records, preparing meals, and traveling out of the neighborhood, on a 0 to 3 scale, with higher scores indicating higher dependency. QoL was assessed using the Quality of Life-AD (QoL-AD) questionnaire (range 13–52) [35]. Thirteen items, such as interpersonal relationships, health, mood, and ability to participate in meaningful activities, were assessed on a 4-point scale, ranging from 1 (poor) to 4 (excellent). Patients’ QoL was assessed twice, both self-rated and caregiver-rated. The weighted QoL-AD composite score is calculated by combining these two scores, with the patients’ QoL score weighing twice as much as the caregiver-rated QoL score [35]. Caregiver burden was assessed with the Zarit Caregiver Burden Interview (ZBI, range 0–88) [36]. Scores between 0 and 20 indicate little to no burden, 21 to 40 indicating mild to moderate burden, and 41 to 88 indicating severe burden. In this study, informant-based questionnaires were filled in by partners of the patient (*n* = 87), children of the patient (*n* = 9), or other informants (*n* = 4).

### Biological measurements

#### Blood and CSF

Blood (serum and plasma) and CSF were collected and stored in our biobank at the Department of Clinical Chemistry of the Amsterdam University Medical Centers Amsterdam, according to the international consensus standard operation procedures [37]. CSF Aβ42, Tau, and pTau concentrations were measured using a sandwich ELISA (Innotest, Fujirebio, Gent, Belgium), or the Elecsys Aβ42, Tau, and pTau (181P) CSF assays run on the *cobas e*601 analyzer (Roche Diagnostics GmbH). For Innotest values, drift-corrected continuous Aβ42 concentrations were used [38]. Concomitant AD pathology was defined as a ratio of pTau/Aβ42 > 0.054 (Innotest) or pTau/Aβ42 > 0.020 (Elecsys) [39].

Apolipoprotein (APOE) ε4 genotype was determined using the LightCycler ApoE Mutation Detection Kit (Roche Diagnostics, GmbH, Mannheim, Germany), after DNA isolation from 10-ml EDTA vacutainer tubes. Patients were dichotomized as APOE-ɛ4 carrier (hetero- and homozygous) or non-carrier.

#### MRI

MRI scanning was performed according to the standardized ADC protocol [10]. Data were acquired using multiple scanners (1.5 and 3 T). Visual assessment of atrophy and cerebrovascular abnormalities was performed by experienced neuroradiologists [10]. Medial temporal lobe atrophy (MTA) was rated using coronal T1-weighted images on a 5-point scale (0–4) [40]. We used the average score of left and right for analysis. Global cortical atrophy (GCA) was rated on FLAIR images using a 4-point scale (0–3) [41]. Posterior cortical atrophy (PCA) was rated on T1-weighted and FLAIR-weighted images in sagittal, axial, and coronal planes, with an average of the left and right scores (range 0–3) [42]. White matter hyperintensities were rated on axial FLAIR images using the Fazekas Scale (range 0–3) [43]. The number of microbleeds was dichotomized as present or not (0–1) [10, 44].

#### EEG/MEG

EEGs were recorded as standard screening of the memory clinic using a digital EEG system and software (Brain RT®; OSG b.v., Rumst, Belgium). The EEG registrations were visually assessed by certified neurophysiologists. In addition to a routine clinical EEG report, EEGs were scored according to a standardized visual rating scheme [45]. From this scheme, we used the 5-point scale to assess the severity of EEG abnormalities (1 = normal EEG, 2 = mildly abnormal, 3 = moderately abnormal, 4 = severely abnormal, 5 = iso-electric EEG; scores 4 and 5 are not expected to be given in an outpatient clinic setting). Severity scores higher than 1 indicated an abnormal EEG. Seven patients had magnetoencephalogram (MEG) available instead of EEG [46]. For MEG, the same 5-point scale was used to assess the severity of abnormalities.

#### Dopamine transporter (DAT) imaging

The SPECT imaging protocol has been described previously in more detail [47]. ^123^FP-CIT SPECT images were visually analyzed by a nuclear medicine physician, using a standard template with five regions of interest of fixed size for the left and right head of the caudate nucleus, left and right putamen, and occipital cortex. Binding ratios (BRs) of specific to non-specific DAT binding were calculated for the left and right putamen and head of the bilateral caudate nuclei, using the occipital cortex as a reference area [47]. The visual assessments as well as age-matched BRs were taken into account in determining whether the scan was normal or abnormal [47].

### Statistical analyses

Statistical analyses were conducted using R version 4.0.2 [48]. To characterize the core and suggestive symptoms, we used descriptive statistics. For all following analyses, dichotomized values for core and suggestive symptoms (absent/present) were used (Table 1). General linear models evaluated the influence of cognition (MMSE) and core (visual hallucinations, parkinsonism, RBD, fluctuations) and suggestive (OH, constipation, micturinal problems, depression, delusions, anxiety, and apathy) symptoms on IADL (FAQ), QoL (QoL-AD), and caregiver burden (ZBI). IADL functioning can also be perceived as a potential determinant of disease burden; therefore, we added FAQ as a putative predictor in the QoL and caregiver burden models as well. First, univariate models were constructed with all core and suggestive features as independent variables and FAQ, QoL-AD, and ZBI as dependent variables in separate models. Subsequently, we constructed multivariate models with backwards selection based on determinants with *p* < 0.10 in the univariate models. All models were adjusted for age, sex, and MMSE. *p* values lower than 0.05 were considered significant.

## Results

Of 100 DLB patient included in DEvELOP, *n* = 10 were female; the average age was 69 ± 6 years, and the mean duration of complaints before baseline visit was 4 ± 3 years (Table 2). The mean MMSE was 25 ± 3, and the mean MoCa was 21 ± 4. A positive family history for dementia was present in *n* = 39 (39%) and for PD in *n* = 15 (15%) patients. ^123^FP-CIT SPECT supported the diagnosis in *n* = 70 (88%) of patients; *n* = 72 (96%) had an abnormal EEG/MEG, of which *n* = 60 (80%) a severity score of 3 (moderately abnormal). Concomitant AD pathology, as defined by an abnormal CSF p-tau/Aβ42 ratio, was present in *n* = 39 (53%) patients. CSF Aβ42 was abnormal in *n* = 51 (70%) cases, while total tau and p-tau were less often abnormal (respectively 37% and 40%). The degree of neurodegeneration on MRI was relatively low; few patients had medial temporal atrophy; the median MTA score was 1 (IQR 0.5–1.5), and the MTA score was ≥2 in *n* = 15 cases (16%). Global cortical atrophy and posterior cortical atrophy score were also low (respectively 1 (IQR 1–2) and 0 (IQR 0–1)). Atrophy scores did not differ between patients with and patients without AD co-pathology in CSF (data not shown). Vascular lesions were infrequent in our cohort (median Fazekas score 1 (IQR 0–1)).

**Table 2:** Baseline characteristics

Domain	Measurement	Total (*n* = 100)	Dementia (*n* = 73)	MCI (*n* = 27)	n available
Demographics	Sex, *n* female (%)	10 (10%)	8 (11%)	2 (7%)	100
	Age, years	69 ± 6	70 ± 5	67 ± 7	100
	Disease duration, years	4 ± 3	4 ± 3	4 ± 2	99
	Education, years	12 ± 3	12 ± 3	12 ± 3	100
	MMSE	25 ± 3	24 ± 3	27 ± 2	100
	APOE-e4 carrier	39 (47%)	26 (47%)	10 (46%)	77
Core clinical features	Parkinsonism	69 (69%)	54 (75%)	15 (56%)	99
	Visual hallucinations	39 (39%)	32 (44%)	7 (27%)	99
	Cognitive fluctuations	45 (46%)	38 (53%)	7 (27%)*	98
	RBD	75 (77%)	55 (76%)	20 (77%)	98
Suggestive neuropsychiatric symptoms	Depressive symptoms	18 (19%)	14 (20%)	4 (16%)	95
	Delusions	7 (7%)	7 (10%)	0	99
	Anxiety	40 (44%)	31 (44%)	9 (41%)	92
	Apathy	56 (61%)	45 (64%)	11 (50%)	92
Suggestive autonomic symptoms	Orthostatic hypotension	63 (64%)	46 (63%)	17 (65%)	99
	Constipation	34 (34%)	29 (40%)	5 (19%)*	100
	Urinary problems	47 (47%)	33 (45%)	14 (52%)	100
	Hynosmia	52 (54%)	40 (56%)	12 (46%)	97
Assessment of disease burden	Functional Activities Questionnaire	12 ± 6	13 ± 6	8 ± 6*	98
	Quality of Life-AD	31 ± 5	31 ± 5	31 ± 5	100
	Zarit caregiver burden interview	25 ± 15	26 ± 14	19 ± 16	77
Imaging	^123^FP-CIT-SPECT, *n* abnormal (%)	70 (88%)	47 (87%)	22 (88%)	80
	Medial temporal atrophy	1 [0.5–1.5]	1 [0.5–1.5]	0.5 [0–1]*	95
	Posterior cortical atrophy	1 [1–2]	1 [1–2]	1 [0.5–1]	95
	Global cortical atrophy	1 [0–1]	1 [0–1]	0 [0–1]*	95
	Fazekas	1 [0–1]	1 [1–1]	0 [0–1]	94
	Microbleeds, presence, *n* (%)	12 (13%)	8 (12%)	4 (17%)	90
EEG/MEG (*n* = 68/*n* = 7)	Abnormal EEG/MEG (severity ≥2)	72 (96%)	52 (98%)	20 (91%)	75
	Severe abnormal EEG/MEG (severity = 3)	60 (80%)	46 (89%)	14 (64%)*	75
CSF	Aβ42, *n* abnormal (%)	51 (70%)	39 (74%)	12 (60%)	73
	t-tau, *n* abnormal (%)	27 (37%)	19 (36%)	8 (40%)	73
	p-tau, *n* abnormal (%)	29 (40%)	20 (38%)	9 (45%)	73
	p-tau/Aβ42, *n* abnormal (%)	39 (53%)	30 (57%)	9 (45%)	73

Data represent *n* (%), mean ± SD, or median [IQR]

*Abbreviations*: *MMSE* Mini-Mental State Examination, *MCI* mild cognitive impairment, *RBD* rapid eye movement sleep behavior disorder, *GDS* Geriatric Depression Scale, *QPE* Questionnaire on Psychotic Experiences, *NPI* Neuropsychiatric Inventory, *NMSS* Non-Motor Symptoms Scale

*p < 0.05 compared to dementia

Seventy-three patients fulfilled the McKeith 2017 criteria for probable DLB; 27 had a diagnosis of MCI-LB. Patients with MCI-LB did not differ from dementia patients on the abnormality of ^123^FP-CIT SPECT scans, EEG scans, or concomitant AD pathology in CSF. MCI-LB patients had less neurodegeneration on MRI, specifically lower MTA and GCA, and lower Fazekas. By nature, cognitive problems and IADL interference were less severe in MCI patients, with lower MMSE scores, less presence of cognitive fluctuations, and lower FAQ scores. Constipation was more frequently present in patients with dementia, compared to patients with MCI. Other clinical symptoms or measures of disease burden did not differ between the MCI and dementia groups.

### Characterization of cognition and clinical symptoms

#### Parkinsonism

*N* = 69 (69%) patients had parkinsonism (bradykinesia with additional rigidity and/or resting tremor). The mean UPDRS score was 20 ± 11 (range 0–53). Postural instability was present in *n* = 52 (52%) of the cases, and *n* = 31 (31%) had gait disturbances. In patients with parkinsonism, the median H&Y stage was 2 (IQR 1–2). In these patients, *n* = 18 (26%) had H&Y stage 1 or stage 1.5, indicating unilateral involvement (with or without axial involvement). Fifty patients (72%) had H&Y stage 2 or higher, indicating bilateral motor symptoms.

#### RBD

Recurrent dream enactment behavior was reported in *n* = 75 (77%) patients and was the most frequently reported core clinical symptom in our cohort. The median duration of RBD symptoms before baseline visit was 4 years (range 3 months up to 40 years, IQR 1.5–10 years). In *n* = 21 (28%) cases with suspected RBD, the patient had injured his/herself due to these symptoms, and *n* = 26 (35%) bedpartners reported that they had been injured because of RBD symptoms.

#### Cognitive fluctuations

*N* = 73 (75%) patients answered positively on at least one of the CAF screening questions, and *n* = 45 (46%) had a total CAF score of ≥5, indicative of cognitive fluctuations. In patients who answered positive on one of the screening questions, the symptoms occurred on a weekly to daily basis in *n* = 33 (46%) and more than daily in *n* = 26 (36%) patients. Caregivers indicated that fluctuations would last for 5 min to an hour in *n* = 28 (38%) of patients and less than 5 min in *n* = 26 (36)% of patients. Daytime somnolence was reported in *n* = 58 (59%) patients. Twenty-five patients (25%) slept for two or more hours during the day on a regular basis. Illogical thinking was reported in *n* = 68 (69%) patients and staring spells in *n* = 59 (60%) patients.

#### Visual hallucinations

Thirty-nine (39%) patients had experienced hallucinations in the past month. Visual hallucinations caused minimal or no distress in most patients (*n* = 30, 77%). The content of the visual hallucinations was valued as positive or neutral by *n* = 32 (82%) patients, while *n* = 7 (18%) sometimes experienced negative content in the hallucinations. When assessing the content of the hallucinations, *n* = 25 (64%) patients reported that they saw complex images such as persons or animals, *n* = 7 (18%) patients reported that they perceived faces or shadows, while the other patients perceived inanimate objects like buildings, or simple forms like circles or flashes of light. Nearly all patients were convinced that the visual hallucinations were not real (*n* = 35, 90%). In the past month, *n* = 27 (27%) patients had experienced visual illusions, and *n* = 25 (25%) patients had experienced passage hallucinations. The feeling of presence was reported by *n* = 23 (23%) patients.

#### Supportive neuropsychiatric symptoms

Auditory hallucinations were present in *n* = 8 (8%) patients, of which *n* = 6 (75%) patients reported that this caused no or minimal distress. The auditory hallucinations contained verbal content (for example, voices, sentences) in *n* = 4 (50%) patients and non-verbal content (for example, music, doorbells) in the other *n* = 4 patients. Three patients reported tactile hallucinations, and one had olfactory hallucinations. All patients who reported hallucinations in non-visual modalities also had visual hallucinations. Delusions were present in *n* = 7 (7%) patients. Four patients had experienced Capgras syndrome, one patient had guilt delusions, two had paranoid delusions, and one had somatic delusions. The mean GDS was 4 ± 3, and *n* = 18 (19%) had a score higher than 5, indicative for depression. Apathy was reported in *n* = 56 (61%) patients and anxiety in *n* = 40 (44%) patients. Of note, we found discordant information on hallucinations in the NPI and QPE in 27% of cases. The NPI identified fewer patients with hallucinations (*n* = 33) compared to the QPE (*n* = 42 in all modalities including visual illusions and passage hallucinations).

#### Supportive symptoms of autonomic dysfunction

Orthostatic hypotension (OH) was present in *n* = 63 (64%) patients, of which *n* = 46 (73%) had delayed and/or prolonged OH (OH after three minutes standing). Sixty percent of patients were symptomatic, of which 36% reported that these complaints were causing moderate or severe distress. Constipation was reported in *n* = 34 (34%) patients in the past month, causing moderate or severe distress in 58% of these patients, while the others report only mild disturbances. Micturition problems were reported in *n* = 47 patients (47%), of which *n* = 27 (57%) reported moderate or severe disturbances. Sexual dysfunction was reported in 29% of patients.

#### Neuropsychological assessment

**Fig. 1:**
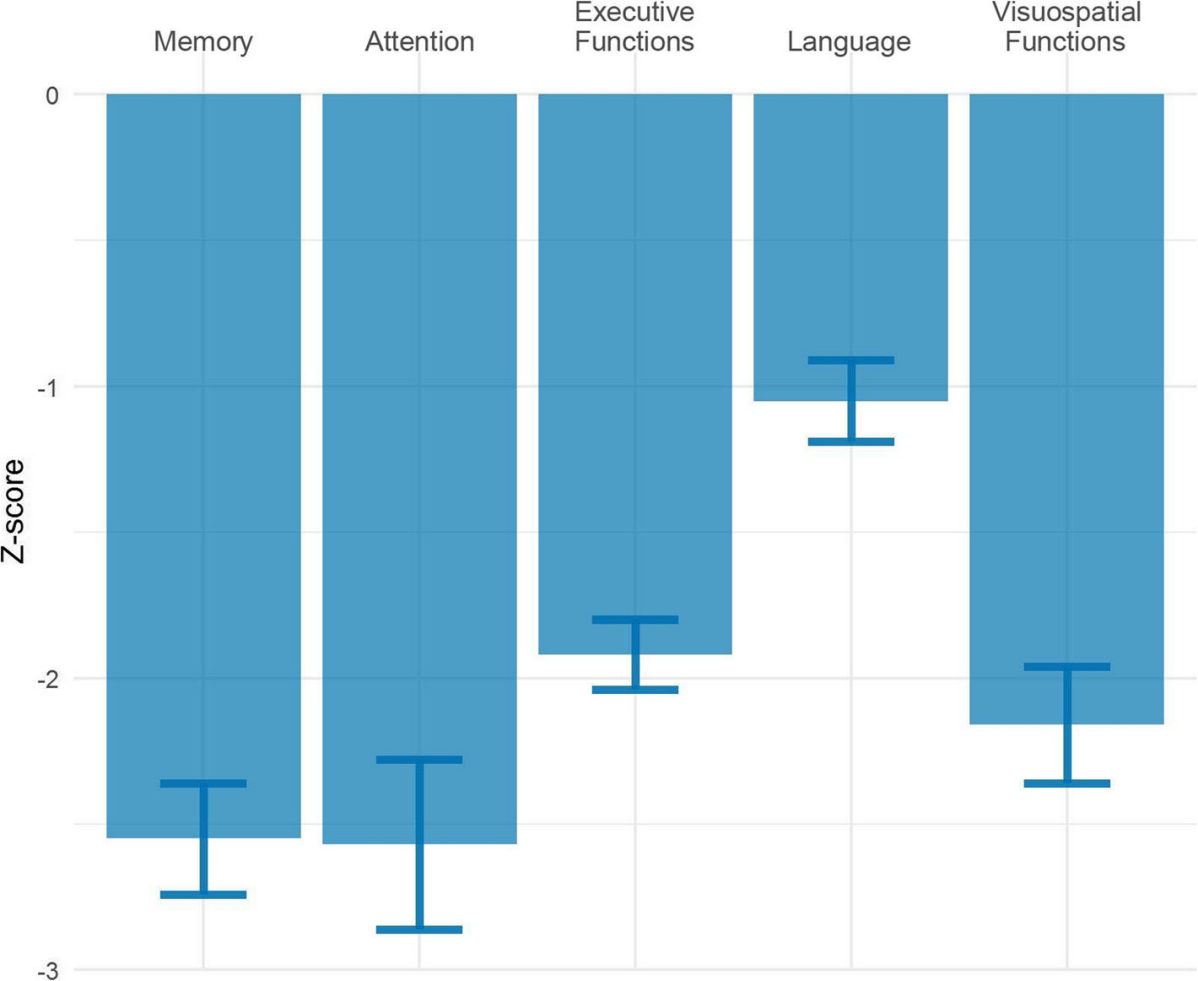
Neuropsychological test results. Bars represent the mean *z*-scores based on data from cognitively healthy subjects. Error bars represent standard error

The neuropsychological domain scores are displayed in Fig. 1. Patients scored lowest in the memory and attention domain, followed by visuospatial functioning. Language was least affected (see Supplementary Table 1 for the complete neuropsychological test battery and raw test results).

### Associations between clinical symptoms and measures of disease burden

We evaluated the associations between core and suggestive symptoms and FAQ (IADL), QoL-AD (QoL), and caregiver burden (ZBI) (Table 3). In univariate analyses, we found that presence of hallucinations, parkinsonism, cognitive fluctuations, apathy, and lower MMSE were associated with lower FAQ scores, indicating lower IADL functioning. In a multivariate model, cognitive fluctuations (*β* ± SE = 4.40 ± 1.12, *p* < 0.001) and lower MMSE (*β* ± SE = − 0.60 ± 0.20, *p* < 0.05) were the strongest predictors of lower FAQ scores, followed by parkinsonism (*β* ± SE = 2.29 ± 1.23, *p* < 0.1), and apathy (*β* ± SE = 2.11 ± 1.10, *p* < 0.1). Using univariate models with QoL as an outcome measure, we found visual hallucinations, constipation, depressive symptoms, and higher IADL dependency were related to lower QoL. In the multivariate model, depressive symptoms (*β* ± SE = − 4.25 ± 1.29, *p* = 0.001), constipation (*β* ± SE = − 2.16 ± 1.07, *p* < 0.05), and IADL functioning (*β* ± SE = − 0.17 ± 0.08, *p* < 0.05) remained significant determinants of QoL. Apathy (*β* ± SE = 11.01 ± 2.79, *p* < 0.001) and higher IADL dependency (*β* ± SE = − 1.07 ± 0.21, *p* < 0.001) were related to higher caregiver burden on ZBI in both the univariate and multivariate analyses.

**Table 3:** Linear regression models of cognition, core and suggestive features, and disease burden

Symptom	FAQ		QoL-AD		ZBI	
	Model 1	Model 2	Model 1	Model 2	Model 1	Model 2
Visual hallucinations	2.48 ± 1.21*		−1.47 ± 1.08		−2.30 ± 3.50	
Parkinsonism	3.42 ± 1.34*	2.29 ± 1.23^+^	−1.79 ± 1.19		6.28 ± 3.85	
RBD	2.07 ± 1.39		0.67 ± 1.24		4.33 ± 4.09	
Cognitive fluctuations	4.75 ± 1.15*	4.40 ± 1.12*	−1.46 ± 1.10		5.73 ± 3.61	
Orthostatic hypotension	0.49 ± 1.26		−1.16 ± 1.11		0.39 ± 3.59	
Constipation	0.74 ± 1.36		−3.09 ± 1.15*	−2.16 ± 1.07*	5.37 ± 3.96	
Urinary problems	−0.92 ± 1.19		0.55 ± 1.04		−4.61 ± 3.34	
Depression	1.12 ± 1.58		−4.64 ± 1.31*	−4.25 ± 1.29*	7.02 ± 4.31	
Apathy	2.87 ± 1.20*	2.11 ± 1.10^+^	−1.06 ± 1.09		13.59 ± 3.11*	11.01 ± 2.79*
Anxiety	1.19 ± 1.22		−0.73 ± 1.08		5.42 ± 3.32	
MMSE	−0.94 ± 0.21*	− 0.60 ± 0.20*	0.15 ± 0.19		−1.12 ± 0.60^+^	
IADL (FAQ)	**–**	**–**	−0.20 ± 0.09*	−0.17 ± 0.08*	1.29 ± 0.25*	−1.07 ± 0.21*

Data represent *β* ± SE. Model 1: with correction for age, sex, and MMSE. Model 2: after backwards selection with significant determinants of model 1, corrected for age, sex, and MMSE

*Abbreviations*: *FAQ* Functional Activities Questionnaire (higher scores indicate lower IADL functioning), *QoL-AD* Quality of Life-Alzheimer’s Disease (higher scores indicate higher quality of life), *ZBI* Zarit Caregiver Burden Interview (higher scores indicate higher caregiver burden), *RBD* rapid eye movement sleep behavior disorder, *QPE* Questionnaire on Psychotic Events, *NPI* Neuropsychiatric Inventory, *UPDRS* Unified Parkinson’s Disease Rating Scale, *MMSE* Mini-Mental State Examination

*p < 0.05

^+^*p* < 0.1

## Discussion

In this paper, we presented the design and baseline results of the DEvELOP cohort. We provided an in-depth characterization of clinical symptoms, of which RBD was the most frequently presented core feature and visual hallucinations were the least frequently reported. Suggestive symptoms such as autonomic dysfunction and neuropsychiatric symptoms were common, in up to 65% of patients. We demonstrated that suggestive symptoms, neuropsychiatric symptoms in particular, were associated to lower QoL and higher caregiver burden, while motor and cognitive symptoms were associated to lower IADL functioning.

The DEvELOP cohort consists of DLB patients with mild to moderate cognitive dysfunction, with the lowest scores in the attention and memory domain compared to other cognitive domains. More than half of the patients had concomitant AD pathology in CSF, which is slightly higher compared to other studies investigating AD co-pathology in CSF [49, 50], but lower compared to pathological studies [51, 52]. This can be due to the differences in the definition of AD pathology based on CSF markers; we used the p-tau/Aβ42 ratio, which is probably the most sensitive for AD pathology [39]. Misdiagnosis is not likely given the high rate of positive ^123^FP-CIT-SPECT scans. The discrepancy between the prevalence of AD pathology in vivo and postmortem suggests that AD pathology develops during the course of the disease in DLB. The degree of atrophy on MRI was relatively mild, in line with previous research investigating atrophy in DLB [53]. A potential explanation for this finding is that many patients in our cohort were in an early disease stage, and neurodegeneration may follow in a later stage [54].

Of all four core symptoms, RBD was most frequently reported and was present up to more than 10 years before diagnosis. This high prevalence and early onset of RBD symptoms are also known from previous literature [55, 56]. More than two thirds of our cohort had signs of parkinsonism, but the degree of parkinsonism was mostly mild and symmetric, as reflected by low UPDRS and H&Y scores. Visual hallucinations were reported in less than half of the patients and were usually complex in nature; patients often reported seeing animals or people. Remarkably, in the majority of patients, visual hallucinations did not lead to distress or impairment. The prevalence of visual hallucinations in our cohort is lower compared to other studies investigating DLB; a recent meta-analysis reported a pooled prevalence of 62% of DLB patients with visual hallucinations [57]. A potential explanation for this discrepancy is that our cohort is set in a memory clinic, and patients were in mild to moderate disease stages. A relevant finding was that we found a discrepancy in the assessment of the presence of hallucinations between the NPI (caregiver-based) and the QPE (patient-based), where patients more often reported visual hallucinations. It is conceivable that sometimes, caregivers are not aware of the patients’ experiences or patients do not tell about it, possibly because of fear or shame. This is in line with a previous study, that found that 16% of caregivers were unaware of visual hallucinations in the patient [58]. These findings suggest that using only caregiver-based instruments for hallucinations could be insufficient, and patients themselves should be asked about the presence of symptoms.

Prior research indicated that disease burden in DLB is higher compared to AD, with lower QoL, higher IADL dependency, and caregiver burden, but DLB is also associated with higher healthcare costs and earlier nursing home admission [59, 60]. To date, no disease-modifying treatment for DLB exists, but disease burden can be minimized by pharmacological or non-pharmacological symptomatic treatment. In regard to optimizing treatment strategies for DLB, it is important to know which symptoms have the greatest impact on disease burden. To assess the influence of symptoms, we associated clinical symptoms with three functional measures of disease burden: IADL functioning, QoL, and caregiver burden. IADL dependency was associated with reduced motor function and cognitive symptoms (lower MMSE, cognitive fluctuations, and apathy). These findings are in line with previous literature and are intuitive, since complex IADL require motor function and sustained attention [61]. Cognitive fluctuations were also associated with lower QoL, in line with a former study that also found that more severe fluctuations were related to lower IADL functioning and lower QoL in dementia [62]. In line with previous studies, we also found that IADL functioning is not only an outcome of disease burden, but a relevant determinant of QoL and caregiver burden as well [4, 5, 63].

While visual hallucinations were not related to disease burden, other neuropsychiatric symptoms played an important role in the burden for both patients and caregivers. We found that the presence of depressive symptoms was the strongest determinant of lower QoL for the patient, in line with previous research [5]. Compared to AD, patients with DLB have higher depressive symptoms and lower QoL [4, 5]. One potential hypothesis is that DLB patients have better insight into their condition compared to AD patients in the early dementia phases [64]. With respect to the caregiver, their burden was most strongly related to neuropsychiatric symptoms, apathy in particular. With respect to the caregiver, their burden was strongly related to neuropsychiatric symptoms. Previous studies also found an association between caregiver burden and neuropsychiatric symptoms, often more stronger than the association between cognition and caregiver burden [65, 66]. Our results extend on these findings by showing that apathy in particular is of influence on the burden of caregivers. Apathy is frequently encountered in DLB as it is in many other types of dementia [67]. Apathy may cause a burden for caregivers, as it leads to a decrease in engaging in social activities, inequality, and conflicts in relationships [8]. The neurobiological underpinnings of apathy are not completely understood. Our results underline the value of finding treatment options for both cognitive fluctuations and apathy, as they have a considerate impact on disease burden for patients. There is some evidence for the beneficial effect of cholinesterase inhibitors [9].

In our cohort, neuropsychiatric symptoms occurred frequently and had a considerable impact on patients and caregivers both in MCI and dementia patients. This demonstrates the relevance of carefully assessing these neuropsychiatric symptoms. Previous studies also showed that even in the earliest disease stages, neuropsychiatric symptoms can be present [12, 68, 69]. Symptoms are non-specific and are often poorly recognized, especially in the early stages. Many neuropsychiatric symptoms are potentially treatable with pharmacological and non-pharmacological intervention. Proper education about the disease and symptoms could reduce the burden on both patients and caregivers [70]. Physical activity or psychological intervention might reduce depressive symptoms and apathy in DLB [71, 72]. Cholinesterase inhibitors seem to have beneficial effects on several neuropsychiatric symptoms next to cognitive improvement. Other psychotropic agents such as antidepressants and atypical antipsychotics are used with varying effects. One should note that DLB patients are often hypersensitive to antipsychotics; at this point, only quetiapine and clozapine, and pimavanserin seem to be tolerated relatively well [9]. In small studies in AD and PD, treatment of apathy with methylphenidate has shown significant improvement [73, 74]. Obstacles in pharmacological treatment are sensitivity to side effects and poor compliance [75]. Evidence for optimal treatment of neuropsychiatric symptoms in DLB patients is lacking and mostly based on expert opinion. In the absence of disease-modifying therapy, optimization of symptomatic treatment should be pursued, and trials to enhance this are warranted.

We included twenty-seven patients that fulfilled the criteria for MCI-LB. These patients did not differ in the presence of non-cognitive symptoms from DLB patients, except for less frequent constipation. This illustrates that patients experience the burden of symptoms, even when the cognitive decline is not yet at the stage of dementia. This underlines the importance of early diagnosis in Lewy body diseases. It also illustrates the distinction between MCI-LB and DLB is artificial, and a more inclusive term should be used to label these patients despite their cognitive status.

While cognitive function is often used as an outcome measure in dementia syndromes, in DLB, non-cognitive symptoms have a significant impact on both patients and caregivers. These non-cognitive symptoms are potentially amenable to symptomatic treatment strategies. Clinicians dealing with patients with Lewy body diseases should be aware of the wide array of symptoms in these patients, and a multidisciplinary approach is necessary for the management of patients and their relatives. For the assessment of treatment effects as well as disease progression, the development of DLB-specific outcome measures is necessary which comprise also the non-cognitive features of this complex disease.

### Strengths

Strengths of the present study include our standardized assessment of core and suggestive features and relevant biomarkers. DEvELOP follows the JPND guidelines for prospective cohort studies in DLB [15]. Therefore, harmonization and data sharing with other cohorts are possible. In addition, since there is increasing attention for the earliest phases of the disease, we also included patients with MCI to capture a broad spectrum of disease stages. Last, using standardized follow-up with repeated biomarker assessment, we can address future research questions regarding the course of the disease.

### Limitations

Our study has some limitations. First, although all patients are asked for brain donation, there is no pathological data available yet; hence, misdiagnosis cannot be ruled out. Especially in the few patients with normal ^123^FP-CIT-SPECT scans, pathological confirmation is needed. Second, because of non-normally distributed data on clinical symptoms, the regression analyses were performed on dichotomous determinant variables. A drawback of using binary data is that the subtle differences in symptom severity were not taken into account in defining the influence on disease burden. Third, this is a selected cohort of relatively mildly affected patients recruited in a tertiary memory clinic. This could hamper the generalizability of our findings. DLB patients primarily referred to movement disorder or psychiatric clinics or residing in nursing homes could display different clinical profiles, e.g., more frequent hallucinations in a psychiatric setting and more severe parkinsonism in a movement disorders setting. Also, in more advanced stages of the disease, the relationship between clinical symptoms and disease burden could be different. Last, our gender distribution is more skewed than other study reports, with only 10% of our participants being female [2, 76]. We have no clear explanation for this. In a previous study, we found that DLB manifests differently in women than in men, with more severe cognitive impairment and more frequent AD co-pathology in women [77]. It is imaginable that DLB in women is more often not recognized. To control for the influence of sex differences, all analyses were corrected for sex (and age).

## Conclusions

To conclude, we presented a deeply phenotyped DLB cohort and found clinically relevant associations between symptoms and disease burden. This cohort will be a starting point for future studies. With ongoing data collection, we aim to further investigate the specific research questions regarding the biological mechanisms underlying heterogeneity and prognosis over time.

## Supplementary Information

**Additional file 1: Supplementary Figure 1.** Assessment of hallucinations (all domains) with Questionnaire of Psychotic Experience (QPE) versus Neuropsychiatric Inventory (NPI).

**Additional file 2: Supplementary Table 1.** Clinical questionnaires & tests.

## Data Availability

The data and materials used in this study can be made available by the corresponding author upon request.
