# Supplementary figures and images for: Characterization of symptoms and determinants of disease burden in dementia with Lewy bodies: DEvELOP design and baseline results
Source: Alzheimers Res Ther. 2021 Feb 26;13:53. doi: 10.1186/s13195-021-00792-w (PMC7908769; doi:10.1186/s13195-021-00792-w)

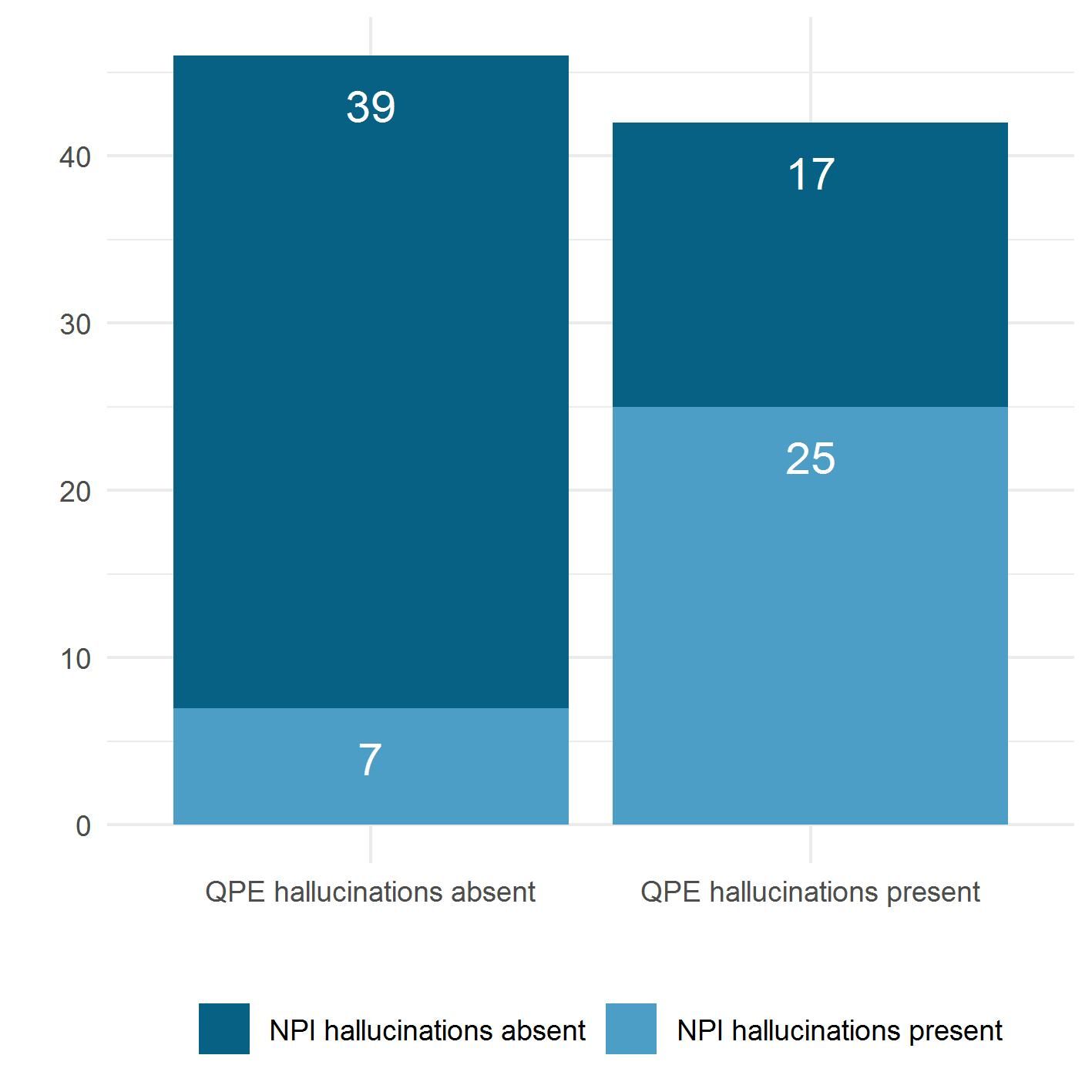

Supplement: Supplementary file 1 — Additional file 1: Supplementary Figure 1. Assessment of hallucinations (all domains) with Questionnaire of Psychotic Experience (QPE) versus Neuropsychiatric Inventory (NPI). [file 13195_2021_792_MOESM1_ESM.jpg]
