# Supplementary material for: Characterization of symptoms and determinants of disease burden in dementia with Lewy bodies: DEvELOP design and baseline results
Source: Alzheimers Res Ther. 2021 Feb 26;13:53. doi: 10.1186/s13195-021-00792-w (PMC7908769; doi:10.1186/s13195-021-00792-w)
Supplement: Supplementary file 2 — Additional file 2: Supplementary Table 1. Clinical questionnaires & tests. [file 13195_2021_792_MOESM2_ESM.docx]

# Supplementary table 1: clinical questionnaires & tests

| Category | Domain | Name of test or questionnaire | n | Range test | Score | Cut-off | # above cutoff |
| --- | --- | --- | --- | --- | --- | --- | --- |
| Clinical evaluation | Anamnesis | Medical history | 100 | - | - |  |  |
|  |  | Family history of dementia | 95 | - | N = 37 |  |  |
|  |  | Family history of Parkinson’s disease | 95 | - | N = 14 |  |  |
| Physical symptoms | Parkinsonism | Unified Parkinson’s disease rating scale (UPDRS) – part 3, motor | 99 | 0-108 | 21±11 | ≥1 on rigidity, bradykinesia and/or resting tremor | 69 |
|  |  | Hoehn & Jahr | 91 | Stage 1, 1.5, 2, 3 | 2 [0-2] | ≥2 | 54 |
|  | RBD | Mayo sleep questionnaire | 98 | 1-4 | 2 [1-3] | ≥1 | 75 |
|  | Orthostatic hypotension | Blood pressure measurement, 3 times | 99 | - | - | ≥20 fall in SBP or 10 in DBP | 63 |
|  | Obstipation | NMSS Q21 - obstipation frequency x severity | 100 | 0-12 | 0 [0-2] | ≥1 | 34 |
|  | Urinary problems | NMSS Q22 – urinary | 100 | 0-12 | 0 [0-4] | ≥1 | 47 |
| Neuropsychiatric symptoms | Visual hallucinations | QPE - modality visual hallucinations | 99 | 0-24 | 0 [0-4] | Frequency past month ≥1 | 39 |
|  | Auditory hallucinations | QPE - modality auditory hallucinations | 99 | 0-24 | 0 [0-0] | Frequency past month ≥1 | 8 |
|  | Tactile hallucinations | QPE - modality tactile hallucinations | 99 | 0-24 | 0 [0-0] | Frequency past month ≥1 | 3 |
|  | Olfactory hallucinations | QPE - modality olfactory hallucinations | 99 | 0-24 | 0 [0-0] | Frequency past month ≥1 | 1 |
|  | Delusions | QPE - delusions | 99 | 0-10 | 1 [0-1] | Frequency past month ≥1 | 7 |
|  |  | NPI total score | 92 | 0-144 | 15±13 |  |  |
|  | Apathy | NPI – apathy subscale (frequency x severity) | 92 | 0-12 | 2 [0-6] | ≥1 | 56 |
|  | Anxiety | NPI – anxiety subscale (frequency x severity) | 92 | 0-12 | 0 [0-2] | ≥1 | 40 |
|  | Depression | Geriatric depression scale – 15 items | 95 | 0-15 | 4±3 | ≥6 | 18 |
| Cognitive fluctuations |  | Mayo fluctuation scale | 98 | 0-4 | 2 [1-3] | ≥3 |  |
|  |  | Clinical assessment of fluctuations | 98 | 0-16 | 4 [0-8] |  | 45 |
| Disease burden | Quality of Life | Quality of Life AD | 100 | 13-52 | 31±5 |  |  |
|  | Activities of Daily living | Functional activities questionnaire | 98 | 0-30 | 12±6 |  |  |
|  |  | Disability Assessment for Dementia | 89 | 0-100 | 80±16 |  |  |
|  | Caregiver burden | Zarit Caregiver burden interview | 77 | 0-88 | 25±14 |  |  |
| Neuropsychological tests | Global cognition | MMSE | 99 | 0-30 | 25±3 | <26 |  |
|  |  | MoCa | 90 | 0-30 | 21±4 |  |  |
|  | Memory | Dutch RAVLT – immediate recall | 100 | 0-75 | 27±10 |  |  |
|  |  | Dutch RAVLT – delayed recall 20 min | 100 | 0-15 | 4±3 |  |  |
|  |  | Dutch RAVLT - Recognition | 99 | 0-30 | 25±4 |  |  |
|  |  | Visual association test - A - 2 trials | 98 | 0-12 | 9±3 |  |  |
|  |  | Rey complex figure delayed recall | 36 | 0-36 | 11±2 |  |  |
|  | Attention | Digit span forward | 97 | 0-8 | 6±1 |  |  |
|  |  | Trail making test (TMT)-A | 99 | seconds | 90±62 |  |  |
|  |  | Stroop word naming | 94 | seconds | 66±24 |  |  |
|  |  | Stroop color naming | 93 | seconds | 87±27 |  |  |
|  | Executive functioning | Trail making test (TMT)-B | 59 | seconds | 213±104 |  |  |
|  |  | Digit span backwards | 97 | 0-8 | 4±1 |  |  |
|  |  | Stroop color word test | 87 | seconds | 189±80 |  |  |
|  |  | Frontal assessment battery | 92 | 0-18 | 13±3 |  |  |
|  |  | Letter fluency test (version D-A-T) | 95 |  | 27±12 |  |  |
|  |  | WAIS similarities | 91 | 0-38 | 20±6 |  |  |
|  |  | Adaptive digit ordering test (DOT-A) | 92 | 0-12 | 4±2 |  |  |
|  | Language | Category fluency test (animals) | 95 |  | 15±5 |  |  |
|  |  | Visual association test – naming | 98 | 0-12 | 12±2 |  |  |
|  | Visuospatial | Rey complex figure copy test | 44 | 0-36 | 28±7 |  |  |
|  |  | Number location - VOSP | 97 | 0-10 | 8±2 |  |  |
|  |  | Dot counting- VOSP | 88 | 0-10 | 9±1 |  |  |
|  |  | Fragmented letters - VOSP | 93 | 0-20 | 16±4 |  |  |
| Abbreviations: RBD: rapid eye movement sleep behavior disorder; SBP: systolic blood pressure; DBP: diastolic blood pressure; NMSS: non-motor symptoms scale; QPE: Questionnaire on psychotic experiences; NPI: neuropsychiatric inventory; QoL-AD: Quality of Life in Alzheimer’s disease; MMSE: mini-mental state examination; MoCa: Montreal cognitive assessment; RAVLT: Raven auditory verbal learning test; VOSP: Visual object and space perception battery. | | | | | | | |
